# European Bats as Carriers of Viruses with Zoonotic Potential

**DOI:** 10.3390/v6083110

**Published:** 2014-08-13

**Authors:** Claudia Kohl, Andreas Kurth

**Affiliations:** Centre for Biological Threats and Special Pathogens, Robert Koch Institute, Nordufer 20, 13353 Berlin, Germany; E-Mail: kurtha@rki.de

**Keywords:** bats, zoonoses, virus, virus discovery, emerging infectious disease

## Abstract

Bats are being increasingly recognized as reservoir hosts of highly pathogenic and zoonotic emerging viruses (Marburg virus, Nipah virus, Hendra virus, Rabies virus, and coronaviruses). While numerous studies have focused on the mentioned highly human-pathogenic bat viruses in tropical regions, little is known on similar human-pathogenic viruses that may be present in European bats. Although novel viruses are being detected, their zoonotic potential remains unclear unless further studies are conducted. At present, it is assumed that the risk posed by bats to the general public is rather low. In this review, selected viruses detected and isolated in Europe are discussed from our point of view in regard to their human-pathogenic potential. All European bat species and their roosts are legally protected and some European species are even endangered. Nevertheless, the increasing public fear of bats and their viruses is an obstacle to their protection. Educating the public regarding bat lyssaviruses might result in reduced threats to both the public and the bats.

## 1. Introduction

The European continent is inhabited by 52 bat species; some are threatened with extinction on the population level and are hence protected under the International Union for Conservation of Nature (IUCN) Red List of Threatened Species and the Convention on the Conservation of Migratory Species of Wild Animals [1,2]. The European bat species that inhabit colder regions use torpor and hibernation. Many bat species migrate over vast distances while others are rather territorial. All bats in Europe utilize echolocation to navigate. Contrary to the worldwide efforts in protecting bats, they have been increasingly gaining attention as potential reservoir hosts of some of the most virulent viruses we know. Various publications reviewed bats globally as carriers and potential reservoir hosts of human-pathogenic and zoonotic viruses [3,4,5,6,7,8,9,10], while hardly anything is known about human-pathogenicity of European bat viruses apart from lyssaviruses. In this review, we discuss a selection of viruses as possible threats posed by European bats to the public from our point of view. A summary of viruses that have been detected in European bats is given in Table 1 at the end of the manuscript. A more comprehensive and up-to-date list of bat-associated viruses can be found online at the *Database of Bat-associated Viruses* (DBatVir) [11].

European bat lyssaviruses (family *Rhabdoviridae*) are the most important zoonotic bat-borne viruses in Europe and have been comprehensively reviewed by Banyard* et al.* in this special issue on bat viruses (Title: Lyssavirus Infections of Bats: Emergence and Zoonotic Threat) [12]. Therefore, we will provide a short overview.

**Table 1:** Viruses of European Bats.

Virus Family	Genus	Bat Species	Origin	Detection	Refs.
*Adenoviridae*	Mastadenovirus	*Pipistrellus* *nathusii* *Pipistrellus pipistrellus*	Germany	Isolation PCR	[13,14]
		*Nyctalus* *noctula* *Rhinolophus* *ferrumequinum*	Hungary	PCR	[15]
		*Myotis* *myotis*	Germany	PCR	[16]
*Astroviridae*	Mamastrovirus	*Myotis* *myotis*	Germany	PCR	[16]
	Mamastrovirus	*Myotis daubentonii* *Myotis* *bechsteinii* *Plecotus auritus*	Hungary	PCR	[17]
*Borna-viridae*	unclassified	*Myotis nattereri* *Pipistrellus pipistrellus*	France	Metagenomics	[18]
*Bunyaviridae*	Phlebovirus Toscana virus	*Pipistrellus* *kuhlii*	Italy	Isolation	[19]
	Nairovirus	*Myotis mystacinus*	France	Metagenomics	[18]
*Coronaviridae*	Alphacoronavirus	*Myotis bechsteinii* *Myotis* *dasycneme* *Myotis daubentonii* *Pipistrellus* *nathusii* *Pipistrellus* *pygmaeus*	Germany	PCR	[20]
		*Myotis blythii* *Myotis daubentonii* *Myotis myotis* *Mineropterus schreibersii* *Nyctalus lasiopterus* *Pipistrellus kuhlii* *Pipistrellus sp.*	Spain	PCR	[21]
		*Hypsugo savii* *Nyctalus noctula* *Pipistrellus kuhlii* *Pipistrellus spp.* *Rhinolophus hipposideros*	Italy	PCR	[22]
		*Miniopterus schreibersii* *Nyctalus leisleri* *Rhinolophus euryale* *Rhinolophus blasii* *Rhinolophus ferrumequinum* *Rhinolophus mehelyi*	Germany	PCR	[16]
		*Myotis daubentonii* *Myotis nattereri*	United Kingdom	PCR	[23]
	Betacoronavirus	*Miniopterus schreibersii* *Nyctalus leisleri* *Myotis daubentonii* *Rhinolophus* *euryale* *Rhinolophus* *blasii* *Rhinolophus* *ferrumequinumv* *Rhinolophus* *mehelyi* *Rhinolophu*s *hipposideros*	Bulgaria Germany	PCR	[24]
		*Rhinolophus* *hipposideros*	Slovenia	PCR	[25]
		*Pipistrellus* *nathusii*	Ukraine	PCR	[26]
		*Pipistrellus pipistrellus*	Netherlands	PCR	[27]
		*Eptesicus* *isabellinus* *Hypsugo* *savii*	Spain	PCR	[21]
		*Eptesicus serotinus* *Hypsugo savii* *Nyctalus noctula* *Pipistrellus kuhlii* *Pipistrellus sp.* *Rhinolophus hipposideros*	Italy	PCR	[22,28]
*Filoviridae*	Cuevovirus	*Miniopterus* *schreibersii*	Spain	PCR	[29]
*Hepeviruses*	Hep-E-related viruses	*Eptesicus* *serotinus* *Myotis* *bechsteinii* *Myotis* *daubentonii*	Germany Bulgaria	PCR	[30]
*Herpesviridae*	Betaherpesvirus Gammaherpesvirus	*Myotis* *myotis* *Myotis* *nattereri* *Nyctalus* *noctula* *Pipistrellus* *pipistrellus* *Plecotus* *auritus*	Germany	PCR	[31]
	Betaherpesvirus Alphaherpesvirus	*Rousettus* *aegyptiacus*	Hungary	PCR	[15]
	Gammaherpesvirus	*Eptesicus* *serotinus*	Hungary	PCR	[32]
*Papillomavirus*	Papillomavirus	*Eptesicus serotinus* *Rhinolophus ferrumequinum*	Spain	PCR	[33]
*Paramyxoviridae*	Unassigned	*Myotis* *mystacinus* *Nyctalus noctula* *Pipistrellus* *pipistrellus*	Germany	PCR	[34]
	Morbillivirus	*Myotis* *bechsteinii* *Myotis* *daubentonii* *Myotis* *myotis* *Myotis mystacinus* *Myotis* *alcathoe* *Myotis* *capaccinii*	Bulgaria Germany Romania	PCR	[35]
*Reoviridae*	Orthoreovirus	*Myotis* *mystacinus* *Nyctalus noctula* *Pipistrellus* *pipistrellus* *Pipistrellus* *nathusii* *Pipistrellus* *kuhlii* *Plecotus auritus*	Germany	IsolationPCR	[36]
		*Pipistrellus* *kuhlii* *Rhinolophus* *hipposideros* *Nyctalus noctula* *Tadarida* *teniotis* *Nyctalus noctula*	Italy	Isolation PCR	[37]
	Rotavirus	*Myotis mystacinus*	France	Metagenomics	[18]
Retrovirus	Gammaretrovirus	*Eptesicus serotinus*	France	Metagenomics	[18]
*Rhabdoviridae*	Various European bat lyssaviruses	*Eptesicus* *serotinus* *Eptesicus isabellinus* *Hypsugo savii* *Minopterus schreibersii* *Myotis myotis* *Myotis daubentonii* *Myotis dasycneme* *Myotis nattereri* *Plectorus auritus* *Pipistrellus pipistrellus* *Rhinolophus ferrumequinum* *Rousettus aegyptiacus* *Vespertilio murinus* *unclassified Chiroptera*	Denmark France Finland Germany Hungary Netherlands Poland Slovakia Spain Switzerland Ukraine United Kingdom	Microscopy isolation PCR	[33,38,39,40,41,42,43,44,45,46,47,48,49,50,51,52,53,54]

## 2. Molecular Detection of European Bat Viruses

The postulates drafted by Jacob Henle and Robert Koch in the late 19th century constitute a framework regarding the principles of cause-and-effect in microbiology [55]. Back then, it was comparatively straightforward to limit cause-and-effect to four postulates, although viruses had not yet been discovered nor was molecular biology developed (Table 1). All of the postulates are hard to fulfill for viruses, as they do not grow on nutrient media, but require living cells for replication. When looking for viruses on a molecular level, it is necessary to consider that only the first postulate can be accomplished. Studies identifying a host-pathogen relationship solely at the molecular level do not take into consideration that detection does not equal etiology. Even though polymerase chain reaction (PCR) screening and metagenomic studies are indispensable and valuable tools, virologists should stay close to the Henle-Koch postulates when assuming a possible virulence of viruses detected in bat hosts.

### 2.1. European Bat Coronaviruses

A plethora of coronaviruses has been detected in bats, mostly belonging to the *alpha*- and *betacoronaviruses* [11,56]. The genus *alphacoronavirus* hosts human-pathogenic strains (*i.e.*, Human CoV 229E and NL63); however, in this review, we will focus on selected highly human-pathogenic betacoronaviruses and their European bat virus relatives [56].

From November 2002 until July 2003 the world was confronted with the first pandemic of the new millennium, caused by a novel coronavirus (CoV) inducing the *Severe Acute Respiratory Syndrome* in humans (SARS) [57,58,59]. The pandemic spread from its origin, a wet-market in the Guangdong province in China, through 33 countries on five continents resulted in more than 8000 infected humans of whom more than 700 eventually died [60,61]. The search for the animal reservoir began, identifying masked palm civets and bats as possible sources. Subsequently, a plethora of diverse coronaviruses of distinct groups have been detected in various bat species around the world via molecular-biological techniques. In 2012, another human-pathogenic coronavirus, called Middle East respiratory syndrome coronavirus (MERS-CoV), began spreading from the Arabian Peninsula, so far resulting in globally 707 laboratory-confirmed cases of infection with MERS-CoV, including at least 252 deaths [62]. Dromedaries and bats are suspected as reservoirs for MERS-CoV [63]. Recent findings support the plausibility of dromedaries as reservoir species [64].

**Figure 1:**
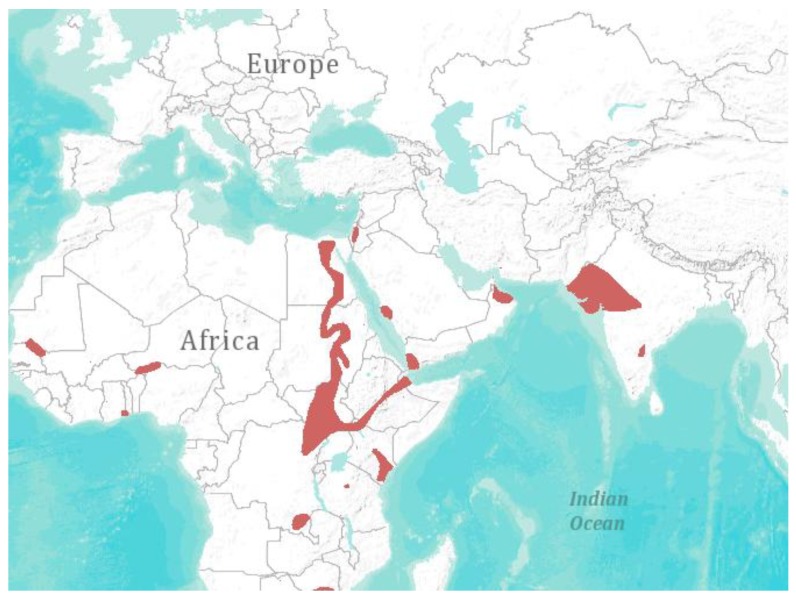
Geographical distribution of* Taphozous perforates* [1]. Visualization: *ArcGIS Explorer.1:50.000.000* [71] (Map sources: Esri, U.S. Geological Survey (USGS), National Oceanic and Atmospheric Administration (NOAA). Sources: Esri, DeLorme, USGS, NPS. Spatial data source: International Union for Conservation of Nature (IUCN))*.*

Although numerous studies in European bats report the presence of SARS-like-CoV and MERS-like-CoV sequences [21,24,25,26,65], no final conclusion can be drawn regarding their zoonotic potential. A related virus detected in bats cannot necessarily be considered as zoonotic. A few alterations in the SARS-CoV spike protein enabled its binding to the host receptor ACE-2, thus SARS-CoV became capable of infecting humans [66]. So far, the SARS-like CoV detected in European bats lack these alterations and thus are not predicted to be capable of infecting humans. Although virus strains might be similar or related on a nucleic acid level, the distinct function of proteins is crucial when determining the host range. Therefore, mere similarity is not sufficient to examine the potential of viruses to infect humans or even predict their virulence. It took ten years from the emergence of SARS-CoV for the first bat CoV to be isolated from *Rhinolophus* bats in China, that displayed the human ACE-2 receptor, which enabled the virus to infect human cells [67]. These findings provide evidence for the reservoir theory. From the European perspective, nevertheless, no SARS-like CoV or MERS-like CoV has been isolated from any European bat, nor has any transmission of SARS-like CoV or MERS-like CoV to humans been reported. The case of MERS-CoV is slightly different, as a sequence of 190 base pairs with 100% identity to MERS-CoV was detected in a bat (*Taphozous perforates*—the species identification performed was not beyond doubt, as it was based on exclusion criteria (no Cytochrome b sequence of *Taphozous perforates* is available in GenBank [68])) in Saudi Arabia [8]. This finding initiated a controversy among leading CoV experts, as the journal *Nature* recently reported [69]. They discussed that the complete genome sequence of MERS-CoV obtained from the bat should confirm that the virus was indeed identical and not coincidentally just a short conserved region of the virus genome. Furthermore, a prevalence study might provide insights into the distribution of MERS-CoV in bat populations. Although *Taphozous perforates* are not abundant in Europe, climate change and environmental factors may have an effect on the future distribution of this bat species (Figure 1) [70]. The case of MERS-CoV emergence impressively demonstrates the necessity of virus discovery and prevalence studies. With the first sequence of MERS-CoV that became available, bats were suspected as reservoir hosts, not only because MERS-CoV is a SARS-CoV relative, but also because previous bat virus discovery studies had provided eligible sequences of bat CoV to GenBank, allowing for correlations with the novel MERS-CoV. Recently, a quasi-species of MERS CoVs was recovered from nasal swabs of dromedaries of the Kingdom of Saudi Arabia [64]. The MERS CoV consensus genome variants from dromedaries and humans are indistinguishable, supporting the plausibility of dromedaries in the role of transmission [64].

### 2.2. European Bat Filoviruses

**Figure 2:**
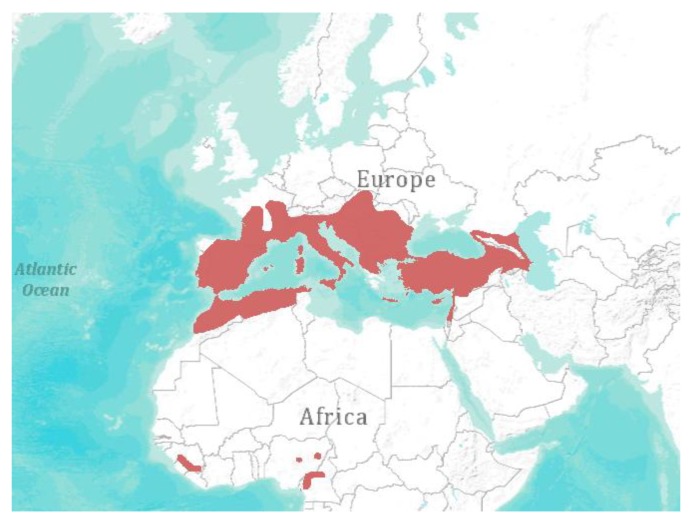
Geographical distribution of Miniopterus schreibersii [1]. Visualization: *ArcGIS Explorer.1:50.000.000* [71] (Map sources: Esri, U.S. Geological Survey (USGS), National Oceanic and Atmospheric Administration (NOAA). Sources: Esri, DeLorme, USGS, NPS. Spatial data source: International Union for Conservation of Nature (IUCN))*.*

In 2002, the first reported outbreak of filovirus, named Lloviu virus (LLOV), in a European bat population occurred in France, Spain, and Portugal [29]. Several colonies of Schreiber’s bats (*Miniopterus schreibersii*) suddenly declined due to an unknown disease. LLOV was found in animals that showed signs of viral infection, but not in healthy bats co-roosting in the caves (*Myotis myotis*). LLOV is distinctly related to filoviruses found in African bats and was classified in 2013 as type species of the novel genus *Cuevavirus* [56]. Unfortunately, the lack of successful isolation of LLOV prohibits the experimental infection of Schreiber’s bats to clarify whether LLOV is the first filovirus capable of inducing disease in bats. This would challenge the hypothesis of bats as potential reservoir hosts for other filoviruses like Ebola and Marburg virus. Schreiber’s bats are distributed in distinct lineages throughout Oceania, Africa, Southern Europe, and South-East Asia (Figure 2) [72]. They are thought to transmit and maintain LLOV across different lineages throughout their habitats, although no studies are available to prove this hypothesis.

Consequently, the sole demonstration of a novel filovirus sequence does not provide evidence of a possible public health threat. Following the Henle-Koch postulates, the virus should be isolated and further characterized to draw conclusions on the evolution of filoviruses in their respective bat host. As most filoviruses are described as highly pathogenic for humans, the occurrence of LLOV should be carefully monitored by prevalence studies in the highly abundant *Miniopterus schreibersii* (Figure 2).

### 2.3. European Bat Paramyxoviruses

In 2012, three distinct paramyxoviruses were detected in German bats, two of which were related to the proposed genus *Jeilongvirus* (*Myotis mystacinus*, *Pipistrellus pipistrellus*) and one was related to the genus *Rubulavirus* (*Nyctalus noctula*) [34]. Another study published in the same year described another 12 different paramyxoviruses in bats from Germany (*Myotis bechsteinii, M. daubentonii, M. myotis*, and *M. mystacinus*) and Bulgaria (*Myotis alcathoe* and* M. capaccinii*), all of which belong to the genus *Morbillivirus* [35].

None of the novel bat paramyxoviruses are closely related to viruses of the highly pathogenic genus *Henipavirus* or other human-pathogenic paramyxoviruses [34,35]. There is no evidence to suggest that any of these novel paramyxoviruses are capable of infecting humans. Similar to the case of the LLOV filovirus, virus isolates and prevalence studies in both humans and bats could improve knowledge and clarify their zoonotic potential.

### 2.4. Other Human-Pathogenic Viruses

Few studies have documented the negative results from PCR testing of European bats for other human-pathogenic viruses. For instance following generic PCR screening for flavi-, hanta- and influenza-A viruses in 210 European bats in 2011 [73], testing of another 1369 Central European bats for influenza-A viruses [74] and testing 42 European bats for hepadnaviruses in 2013 did not lead to the detection of any viral nucleic acids [75]. PCR screening of 468 European bats for orthopoxviruses has not revealed any known or novel virus sequences [76].

## 3. European Bat Virus Isolates

So far, the only virus isolates (beside lyssaviruses) obtained from European bats are one bunyavirus, one adenovirus and 22 orthoreoviruses [13,19,36,37]. These represent the only isolates that would allow for further characterization and potential clarification of their zoonotic potential. Nevertheless, recombinant viruses, constructed on sequence information, are also valuable tools to study prevalence and pathogenicity* in vitro*.

### 3.1. European Bat Bunyavirus Isolate

Toscana virus (TOSV) was isolated from a bat’s brain in 1988, while simultaneously TOSV was isolated from sandflies in the laboratory [19]. As TOSV has never been reported in bats afterwards and no hemagglutination-inhibiting antibodies has been initially found in the bat, there is a reasonable chance that this TOSV isolation may have been a cross-contamination [77].

### 3.2. European Bat Adenovirus Isolate

Bat adenovirus 2 (Bat AdV-2) was isolated from a bat’s intestine in 2009 [13], and the whole genome was obtained and circumstantially analyzed [14,78]. Bat AdV-2 displays a monophyletic relationship to the adenoviruses of canids (CAdV). Moreover, open trading frames (ORF) in the Bat AdV-2 genome and the CAdV are identical and not present in other members of the mastadenoviruses. The closely related canine AdV contribute to the severe kennel cough syndrome in canids and show an unusually broad host range [79]. This provides evidence suggesting an ancestral inter-species transmission of mastadenoviruses between bats and canids. Like in the case of rabies virus, which is prevalent in both bats and terrestrial mammals (e.g., dogs, raccoons, skunks, and foxes) of the Americas, a continuing exchange and transmission between bats and canids or other terrestrial animals might be possible [80]. There is no evidence of a zoonotic potential of Bat AdV-2.

### 3.3. European Bat Reovirus Isolates

In 2012, three novel orthoreoviruses were isolated from *Plecotus auritus* and* Myotis mystacinus* in Germany [36]. A subsequent PCR screening obtained identical viral sequences also in other bat species: *Pipistrellus pipistrellus*, *Pipistrellus nathusii*, *Pipistrellus kuhlii,* and *Nyctalus noctula*. At the same time, a group in Italy detected further 19 orthoreoviruses in *Myotis kuhlii*, *Rhinolophus hyposideros*, *Tadarida teniotis,* and *Vespertilio murinus* [37].

Summing up the data for the reovirus isolates from Germany and Italy, a close relationship was revealed to the genus *Mammalian Orthoreovirus* (MRV), in particular to an orthoreovirus obtained from a dog (strain T3/D04) with hemorrhagic enteritis in Italy [36,37,81]. No ancestral relationship was assumed here, but rather an opportunistic “behavior” of the novel closely related MRVs, as they were detected in various different bat species. Moreover, the newly isolated MRVs are phylogenetically related to viruses capable of inducing severe meningitis in humans [82]. Recently, a study published by Steyer* et al.* described the detection of an MRV from a child hospitalized with acute gastroenteritis in Slovenia [83]. The causative agent was determined to be an MRV with the highest similarity of 98.4%–99.0% in the respective segments to a bat MRV (T3/Bat/Germany/342/08) [83]. This might indicate a human-pathogenic potential of strain T3/Bat/Germany/342/08. As the case of SARS-CoV has shown that even small changes in the genome are important for determining the host range, this has to be determined for the bat MRVs in further studies. Interestingly, no contact was reported between the infected child and bats, but contact to a domestic dog was assumed [83]. The isolated viruses will allow for a seroprevalence study (cross-reactivity and cross-neutralization with other strains) in humans, which shall be initiated to examine the prevalence of specific antibodies to Bat MRVs in Germany and Italy (where these viruses have been found) to clarify their zoonotic potential. This is especially interesting as Asian bat orthoreoviruses of the genus *Pteropine Orthoreovirus* have already been linked to potentially zoonotic respiratory diseases in humans [84,85].

### 3.4. European Bat Lyssaviruses

Rhabdoviruses of the genus *Lyssavirus* that have been detected in Europe are considerably harmful and truly zoonotic agents, inevitably causing the death of unvaccinated humans if not treated in time before onset of the rabies disease [86]. Even though bat-transmitted lyssaviruses have a fatality rate of virtually 100% and are suspected to be transmissible by bat biting and scratching, the reported total number of human fatalities in Europe is low (*n* = 2–5 since 1963) [86,87,88]. All described hosts of European bat lyssaviruses (EBLV-1 and EBLV-2) are synanthropic, hence sharing their habitats with humans [87]. EBLV-1 has been predominantly detected in *Eptesicus serotinus* and *E. isabellinus* in Europe, both living in buildings, roofs, and attics usually in the southern regions of Europe (*E. serotinus* until 55° North, *E. isabellinus* in southern Portugal—*E. isabellinus* is a North African population of *E. serotinus* that is controversially but not concludingly discussed as a novel species [1]), and male bats are reported to co-roost with multiple bat species [90]. EBLV-1 was also detected in *V. murinus*, *M. schreibersii*,* M. myotis*,* M. nattereri*,* R. ferrumequinum*, and *T. teniotis*. Whether these bat species constitute accidental hosts infected by spillover from co-roosting *E. serotinus* species, or whether they are additional reservoirs, has not yet been determined [38,39,40,41,91].

Two human cases described by Johnson* et al.* were confirmed as infected with EBLV-2, which is prevalent in European *M. daubentonii* and *M. dasycneme* [40,86]. *M. daubentonii* is prevalent in north-eastern Europe and is frequently found co-roosting with *P. pipistrellus* and *M. nattereri*, whereas *M. dasycneme* is found throughout Europe and in the Mediterranean, co-roosting with *M. capaccinii*. So far, none of the co-roosting bats have been reported to carry EBLV-2 [90]. However, spillover transmission to other animals (stone-marten, sheep, and cat) was described for EBLV-1 [92,93,94].

Overall, lyssaviruses prevalent in European bats pose a risk to public health, and preventive measures have already been implemented by many European countries for decades (e.g., surveillance, vaccination plans, and post exposure prophylaxis) [87]. Especially the high-risk occupational groups (*i.e.*, bat workers, bat carers in bat bat hospitals) are at increased risk. However, lyssavirus prevalence in European bats is very difficult to determine and results are very heterogenic [40]. The lyssavirus prevalences are considerably low, but changes of behavior as a result of a lyssavirus infection may be more likely to bring bats into contact with humans. However, it is necessary to balance the risk with the total number of fatal human cases during the last 35 years (five cases in 590 million people living in greater Europe) [87]. Accordingly, the risk is relatively low and would probably fall to zero if people were educated appropriately. Direct contact (bites and scratches) with certain bat species might be risky and require post exposure prophylaxis. Only few of the European bat species are known to be reservoirs of EBLV-1 and EBLV-2, but all of the European species are endangered or close to extinction. Relocation or culling of bat colonies, in spite of being an obvious solution from the viewpoint of the general public, increases the risk of lyssavirus exposure and transmission and should not be considered [95]. Only education can channel public fear to avoid further threats to the bats and the general public.

## 4. Spatial Abundance and Biodiversity

Alexander von Humboldt discovered the latitudinal gradient in species diversity as early as 1799 [96]: The richness of species is subject to a global diversity gradient, abating from the species-rich tropics toward the higher latitudes [97]. Bats influence this gradient significantly. More than 1100 bat species have been described worldwide. Although they are abundant worldwide except for the polar regions, a steep diversity gradient is present from the tropics towards the poles [97,98,99,100]. Are fewer viruses prevalent in European bats because of the lower abundance of species in the more temperate Europe? And is the zoonotic risk posed by bats decreased accordingly?

Only few studies on the biogeography of microorganisms are available. These studies indicate that the latitudinal diversity gradient has either no or a top-down effect on microbial diversity [101,102,103,104,105]. Two studies hypothesized that the local diversity and dispersal of viruses is very high, though overall, the viral diversity is limited on the global scale [106,107]. Therefore, no assumptions can be made regarding the viral diversity in species abundant in temperate climates. As the total number of abundant species might not be essential, the change in biodiversity might play a role.

The effect of decline in biodiversity on the emergence of diseases is subject of numerous publications [108,109,110,111,112,113,114]. Basically, there are arguments in favor of two controversial theories; reduced biodiversity could either increase (dilution effect) or decrease the risk of disease transmission. For almost half of the zoonotic diseases that have newly emerged by spillover since 1940, a preceding change in land-use, agriculture, and wildlife hunting was reported [108]. All of the above-mentioned effects contribute to changes in biodiversity and increased contact situations between human and animal hosts, also in Europe. Once spillover in novel hosts has occurred, a high density of the novel host population eventually facilitates the establishment in the novel niche. Thus, human overpopulation and a decreased biodiversity might be mutual factors promoting the establishment of emerging infectious diseases.

In conclusion, the Baas Becking hypothesis from 1932 might still be appropriate: “Everything is everywhere, but the environment selects” [115].

## 5. Conclusive Remarks

Until now, lyssaviruses have been the only proven zoonotic viruses in European bats and may cause rabies in humans. However, only few bat species are known to transmit lyssaviruses in Europe, and the number of human cases is rather low. Nevertheless, education of the general public should be intensified to avoid easily preventable infections. Although viruses with zoonotic potential have been detected in European bats, no clear assumption can be made without further studies. Sero-prevalence studies should be conducted on the orthoreoviruses isolated from European bats, especially as a closely related virus was detected in a diseased child in Slovenia [83]. Other bat viruses detected by using molecular techniques should be isolated (e.g., MERS-like CoV or Bat Bunyavirus) to allow for characterization and follow-up sero-prevalence studies.

In general, bats are special reservoir hosts because of their biological features, long-time co-evolution and high diversity of viruses that can be found. Furthermore, there is neither a clearly decreased risk in the emergence of zoonotic viruses in temperate climates compared to the tropics nor a decreased risk in regions of lower biodiversity.

In conclusion, many drivers of emergence in the tropics also have validity in Europe. However, European bats are endangered species, and some of them are threatened by extinction. Although lyssaviruses are prevalent in European bats, and some viruses might have a zoonotic potential, the overall hazard for humans is comparably low. Moreover, the protection of bats (and any wildlife) will consecutively also protect the general public.
